# Higher surgeon volume reduces early failure in first time revision of non‐infected total knee arthroplasty: An analysis using data from the United Kingdom National Joint Registry

**DOI:** 10.1002/ksa.12690

**Published:** 2025-05-12

**Authors:** Alexander H. Matthews, William K. Gray, Jonathan P. Evans, Jonathan T. Evans, Sarah E. Lamb, Andrew Porteous, Tim Briggs, Shiraz A. Sabah, Abtin Alvand, Andrew D. Toms, Andrew J. Price

**Affiliations:** ^1^ Getting It Right First Time programme, NHS England London UK; ^2^ Royal Devon University Healthcare NHS Foundation Trust Exeter UK; ^3^ University of Exeter Exeter UK; ^4^ Nuffield Department of Orthopaedics, Rheumatology, and Musculoskeletal Sciences University of Oxford Oxford UK; ^5^ University of Exeter College of Life and Environmental Sciences: University of Exeter Faculty of Health and Life Sciences; ^6^ North Bristol NHS Trust Bristol UK; ^7^ Royal National Orthopaedic Hospital, Stanmore London UK; ^8^ Nuffield Orthopaedic Centre Oxford UK

**Keywords:** mortality rates, re‐revision rates, revision total knee replacement, total knee arthroplasty, volume‐outcomes

## Abstract

**Purpose:**

Revision total knee replacement (RevKR) is an increasingly common procedure. It is hypothesised that higher surgical volume is linked to lower levels of adverse outcomes. The aim was to estimate the association of surgical volume on patient outcomes following first single‐stage RevKR for non‐infected indications.

**Methods:**

This population‐based cohort study used data from the United Kingdom National Joint Registry, Hospital Episode Statistics and National Patient Reported Outcome Measures. Patients undergoing procedures between 1 January 2009 and 30 June 2019 were included. The primary outcome measure was re‐revision within 2 years; chosen to reflect the quality of the surgical provision. Fixed effect multivariable regression models were used to examine the association between surgeon and surgical unit annual caseload and the risk of adverse outcomes.

**Results:**

A total of 8695 patients underwent first time single stage revision for aseptic loosening, instability, or malalignment across 389 surgical units and 1204 surgeons. Following adjustment for age, gender, ASA grade, year of surgery and operation funder, higher surgeon volume was associated with a lower risk of re‐revision at 2 years. The risk of re‐revision decreased amongst surgeons performing ≥9 annual revisions (OR 0.77, 95% CI 0.62–0.95, *p*‐value = 0.02) compared to those performing <9 annual revisions.

**Conclusions:**

Annual surgeon case volume of ≥9 first single‐stage RevKR for non‐infected indications is independently associated with reductions in early re‐revision. This evidence supports the setting of minimum volume targets to improve outcomes for patients.

**Level of Evidence:**

Level III, retrospective cohort study of prospectively collected data.

**Abbreviations**
AIC: Akaike Information Criterion
ASA: American Society of Anaesthesiologists
BMI: body mass index
CI: confidence intervals
CIPS: continuous inpatient stay
EQ‐5D: EuroQol 5 Dimension
HES: hospital episode statistics
HES APC: hospital episode statistics admitted patient care
IMD: index of multiple deprivation
IQR: interquartile range
MDS: minimum data set
MRC: major revision centre
NHS: National Health Service
NJR: National Joint Registry
OKS: Oxford Knee Score
OR: odds ratio
PAU: primary arthroplasty unit
PROMs: Patient Reported Outcome Measures
RCS: restricted cubic splines
RevKR: revision knee replacement
RU: revision unit
TKR: total knee replacement

## INTRODUCTION

There were 5783 revision knee replacements (RevKR) recorded on the United Kingdom National Joint Registry in 2023 [18]. While the number of revisions has remained stable since 2021, RevKR represents a significant burden for the National Health Service (NHS). RevKR has been recognised as a technically demanding procedure with a 49% increased surgical time compared with primary TKR [5]. An increasing revision burden carries significant implications for healthcare systems both clinically and financially [13]. There is a need to understand factors which influence outcomes to help optimise current management strategies.

RevKR services in the UK have recently undergone major reconfiguration to form revision networks [12]. Fewer expert centres and surgeons defined by those with higher annual revision caseloads aim to deliver revision knee replacement based on evidence that this will lead to better results for patients and cost‐efficiencies [3]. An important assumption underpinning the set‐up of revision networks is that a volume‐outcome relationship exists; that is, higher volume surgical units and surgeons achieve better patient outcomes. However, a recent systematic review and meta‐analysis showed very low confidence in the current cumulative evidence for the existence of a volume outcome relationship after RevKR [16]. Given the heterogeneity of procedures that fall under the definition of a revision knee replacement, studies aiming to define links between procedural volume and outcomes require more specific definitions of the patient cohort being investigated. A recent study by the same authors showed higher hospital volume is associated with reductions in re‐revision rates in the more complex scenario of an infected revision knee replacement [15]. However, whether the same relationship exists in a technically simpler revision procedure remains uncertain.

Therefore, the primary aim of the present study was to establish the relationship between procedural volume and re‐revision rate following first, single stage RevKR for non‐infection. The relationship between procedural volume and secondary outcomes such as mortality; serious medical complications within 90 days; knee function and quality of life at six months following revision surgery were also investigated. The null hypothesis was that surgical unit caseload was not associated to the primary or secondary outcomes. The alternative hypothesis was that higher surgical unit caseload was associated to a lower re‐revision rate at two years; a lower mortality rate at 90 days; a lower rate of any serious medical complication at 90 days; improved knee function and quality of life; and reduced length of stay.

## METHODS

### Study design

**Figure 1:**
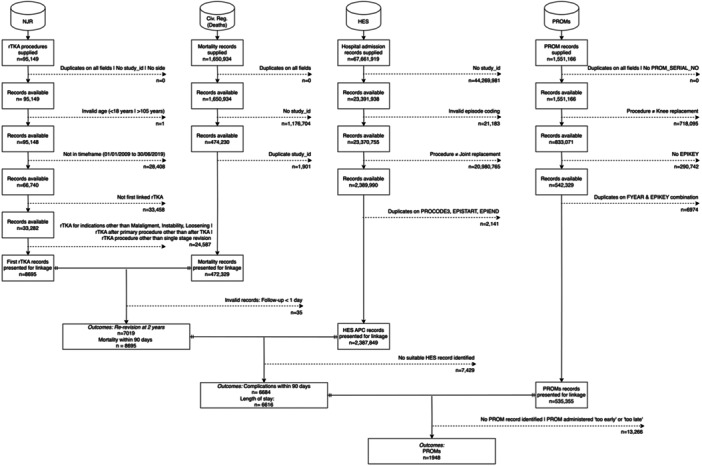
Flowchart demonstrating the attrition of study records during data preparation and record linkage.

In this retrospective observational registry‐based study, data from the National Joint Registry (NJR) was analysed which covers procedures performed at public and private hospitals in England, Wales, Northern Ireland, the Isle of Man and the States of Guernsey. This is the largest registry in the world [18]. This data was linked with other routine health data including Hospital Episode Statistics Admitted Patient Care (HES APC); National Health Service (NHS) Patient Reported Outcome Measures (PROMs); and the Civil Registrations of Death registers. Data cleaning and preparation of the NJR, HES APC data, Civil Registrations of Death and NHS PROMs followed a previously published and reproducible workflow [24]. Data linkage and attrition are summarised in Figure 1. Ethical approval was obtained from the London‐Bromley Research Ethics Committee (20/LO/0428). Data access approvals were obtained from the NJR (RSC2017/26). Patients who did not consent to the NJR audit were not included.

### Participants

All first time, non‐infected, single stage RevKR procedures performed between 1 January 2009 to 30 June 2019 were included. Non‐infection represents the commonest scenario for a RevKR and focussing on these patients helps controls for heterogeneity in presentation [21]. This timeframe was selected (i) to provide a minimum 2‐year follow‐up period to 31 December 2019 (which preceded the Covid‐19 pandemic, where access to RevKR surgery was limited and unlikely to be representative of usual care [8]), and (ii) to exclude early NJR records where compliance with the registry was low. RevKR was defined as “*(any) operation performed to remove (and usually replace) one or more components of a total joint prosthesis for whatever reason*” [17]. Therefore, revisions following partial knee replacement and procedures that could not be linked to a primary TKR in the analysis of unit‐level and surgeon‐level outcomes were excluded. However, these revision procedures did contribute to caseload calculations. *First* RevKR was defined as the earliest revision procedure following a primary total knee replacement recorded in the NJR. Indication for revision was categorised hierarchically into 10 discrete groups: Infection, Malalignment, Loosening/Lysis, Component wear, Instability, Fracture, Progressive Arthritis, Stiffness, Unexplained Pain, Other. This was based on the Australian Orthopaedic Association National Joint Replacement Registry hierarchical system [2]. Where multiple reasons for revision were recorded, the hierarchy was used to determine the primary reason for revision and the revision was attributed to the highest‐ranking reason. In the current study, only procedures revised for Malalignment, Loosening/Lysis, Instability were included. Additionally, all procedures meeting any of the following criteria were excluded: intention to treat in multiple‐stages; amputation, arthrodesis procedures; missing, out of range or invalid entries in key date fields or other fields necessary for case‐mix adjustment.

### Procedures

The exposure of interest was the caseload of each surgical unit and surgeon. Annual caseload was defined as the number of RevKR performed by a surgical unit or surgeon in the 365 days prior to each index procedure. Caseloads were calculated before applying inclusion and exclusion criteria (except for duplicates and unreliable date information) to avoid endogeneity. This meant that RevKR for all indications, re‐revision procedures, revisions of partial knee arthroplasties, and RevKR not linked to a primary TKR were all included. Each stage of a staged procedure was counted separately. For RevKR procedures in 2009, caseloads were calculated using data from 2008 if appropriate. For surgical units newly formed over the study period and new surgeons entering revision practice, RevKR procedures within the first 365 days contributed to caseload calculations, but not to outcome assessment. Earliest recorded revision procedure in each surgical unit and by each surgeon was obtained using an extended NJR data set.

For modelling, surgical unit and surgeon caseloads were converted into mean annual volumes, as described below [27]. The following four groups of known or potential confounding variables were considered in our multivariable logistic regression modelling.

#### Patient factors

Age in years (continuous), gender (male/female), American Society of Anesthesiologists (ASA) grade (1, 2, 3+) obtained from the NJR. Baseline PROM scores (Oxford Knee Score & EQ‐5D) from the linked National PROMs data set when estimating post‐operative PROMs only.

#### Surgical factor

If assessing mean surgeon annual volumes as the primary exposure, then mean surgical unit annual volumes were included as a surgical factor and vice versa.

#### Hospital factor

The NJR collects information on hip, knee, ankle, elbow and shoulder joint replacement surgery performed at both public and private hospitals. Data was collected on whether the operation was funded by NHS providers or independent sector providers. Anecdotal evidence suggests less complex RevKR may be performed in independent surgical sites where access to specialist services such as intensive care facilities is limited.

#### Temporal factor

Calendar year of procedure (2009, 2010, 2011, 2012, 2013, 2014, 2015, 2016, 2017, 2018 and 2019).

### Outcomes

The primary outcome was the rate of re‐revision at 2 years. A two‐year timeframe was selected under the hypothesis that re‐revisions within this *early* period may be more representative of the quality of surgical provision rather than *late* revisions (which might, for example, be due to expected component wear or loosening over time, or infection from a secondary source) [26] This early period also correlates with that reported in the existing literature [24].

Secondary/additional outcomes were:

*90‐day all‐cause mortality*, identified using linked data from Civil Registrations (Mortality) data set;

*Rate of any serious medical complication within 90 days*, identified using the HES APC data set following previously described methodology; [24]

*Post‐operative knee function and health related quality of life* were, identified using the Oxford Knee Score (OKS) [22] and the EQ‐5D index [7] respectively at six months following RevKR from the NHS PROMs data set.

*Inpatient length of hospital admission* identified using the HES APC data set attributed from continuous inpatient spells (CIPS), which is the preferred estimate of length of stay [6]. Length of stay was dichotomised into above vs at or below the median stay.

### Statistical analyses

Mean annual volume was defined as the mean of the caseload across all candidate procedures for a surgical unit or surgeon. This was calculated using group mean centring [27]. Continuous variables were described using means and standard deviations or medians and interquartile ranges (IQR) as appropriate, after visual inspection of data distributions. Categorical data were described using counts with percentages. To summarise the descriptive statistics for surgical unit and surgeon volumes these variables were categorised. Surgical unit caseloads were categorised into four groups (less than 12 cases per year, which corresponds to 1 RevKR per month; 13–24 cases per year, corresponding 1–2 RevKR per month; 25–52 cases per year, corresponding to 3–4 RevKR per month; 53 or more cases per year, corresponding to more than 1 RevKR per week). These volume groups were based on the classification described by Halder et al. [10]. Surgeon volume was summarised in five percentile categories based on a previously reported methodology by Roof et al. [20].

Unadjusted binomial funnel plots demonstrating risk of re‐revision for individual surgeons and units were presented with 95% and 99% control limits. During the modelling of outcomes, where the relationship showed no significant deviation from linearity, continuous variables were modelled as linear terms. Where non‐linearity was found, the model used restricted cubic splines (RCS) when testing associations with outcomes. The Akaike Information Criterion (AIC) was used to select the most parsimonious specification of restricted cubic splines using the final adjusted model. Where non‐linear relationships were investigated using RCS, the results were plotted. Where there appeared to be a single point at which the slope of the regression line changed, change point analysis was conducted using a non‐parametric approach with the cumulative sum test statistic and the additive model of change method.

Fixed effects logistic regression models were used for the outcomes of re‐revisions, serious adverse events (dichotomised into the presence of any medical complications or none), prolonged length of stay and mortality at 90 days. Adjustment for confounding was undertaken incrementally, adjusting for each of the four groups of confounding variables to explore their influence on the volume effect at each stage with reference to model fit statistics. This was done in the following order: patient factors, surgical factors, hospital factors and finally temporal factors. The ultimate decision on the preferred statistical model was assessed using the AIC accepting the model with the lowest AIC. Multicollinearity was assessed using eigenvalues, variance inflation factors and by examination of model parameter estimates with stepwise addition and removal of covariates. Odds ratios with 95% confidence intervals (CIs) and associated *p*‐values were reported. A *p*‐value of <0.05 was taken to indicate statistical significance. Data analysis was performed using R version 4.3.1.

## RESULTS

After applying the eligibility criteria, 33,282 first linked RevKR were recorded between 1 January 2009 and 30 June 2019 for all causes. These included 8695 first, single stage revisions for aseptic reasons. These procedures were performed by 389 surgical units and 1204 surgeons. Descriptive statistics summarising co‐variates by mean annual surgical unit volume and mean surgeon volume are shown in Tables 1 and 2, respectively. A total of 1948 (22.5%) cases had complete linked preoperative and post‐operative PROMs data.

**Table 1:** Descriptive statistics by mean annual surgical unit volume, categorised by a priori categorical variables.

	Mean annual volumes per surgical unit			
	Low	Low‐medium	Medium‐high	High
Mean annual surgical unit volume	≤12	13–24	25–52	≥53
Revisions				
Number of patients	1526	2151	2723	2237
Number of units	208	89	66	19
Number of surgeons	509	439	423	246
Mean annual surgeon volume	7.1 (3.8–12.5)	8.2 (5.2–13)	11 (7.3–17.7)	18.4 (10.4–29.9)
Age (years)				
<60	324 (21.2%)	417 (19.4%)	493 (18.1%)	480 (21.5%)
60–64	187 (12.3%)	298 (13.9%)	340 (12.5%)	274 (12.2%)
65–69	303 (19.9%)	422 (19.6%)	536 (19.7%)	452 (20.2%)
70–74	295 (19.3%)	416 (19.3%)	554 (20.3%)	423 (18.9%)
75–79	234 (15.3%)	330 (15.3%)	428 (15.7%)	349 (15.6%)
80 or more	183 (12%)	268 (12.5%)	372 (13.7%)	259 (11.6%)
Gender				
Female	898 (58.8%)	1249 (58.1%)	1549 (56.9%)	1290 (57.7%)
ASA grade				
ASA1	144 (9.4%)	122 (5.7%)	155 (5.7%)	149 (6.7%)
ASA2	1084 (71%)	1495 (69.5%)	1807 (66.4%)	1555 (69.5%)
ASA3	298 (19.5%)	534 (24.8%)	761 (27.9%)	533 (23.8%)
Year of Surgery				
2009	95 (6.2%)	107 (5%)	127 (4.7%)	138 (6.2%)
2010	89 (5.8%)	148 (6.9%)	190 (7%)	141 (6.3%)
2011	107 (7%)	158 (7.3%)	182 (6.7%)	127 (5.7%)
2012	139 (9.1%)	189 (8.8%)	249 (9.1%)	172 (7.7%)
2013	144 (9.4%)	206 (9.6%)	241 (8.9%)	180 (8%)
2014	135 (8.8%)	212 (9.9%)	254 (9.3%)	188 (8.4%)
2015	181 (11.9%)	229 (10.6%)	290 (10.7%)	231 (10.3%)
2016	179 (11.7%)	255 (11.9%)	314 (11.5%)	250 (11.2%)
2017	181 (11.9%)	276 (12.8%)	341 (12.5%)	327 (14.6%)
2018	183 (12%)	238 (11.1%)	356 (13.1%)	305 (13.6%)
2019	93 (6.1%)	133 (6.2%)	179 (6.6%)	178 (8%)
Operation funder				
Public	1025 (67.2%)	2070 (96.2%)	2671 (98.1%)	2197 (98.2%)
Private	501 (32.8%)	81 (3.8%)	52 (1.9%)	40 (1.8%)

*Note*: Patient level descriptive statistics for single stage first revision total knee replacements for aseptic indications.

Abbreviation: ASA, American Society of Anaesthesiologists.

**Table 2:** Descriptive statistics by mean annual surgeon volume, categorised by a priori categorical variables.

	Mean annual surgeon volume centiles				
	1	2	3	4	5
Mean annual surgeon volume	0–5.4	5.5–8.7	8.8–13.1	13.2–20.2	20.3–50.8
Number of revisions					
Patients	1670	1670	1670	1669	1669
Surgeons	594	225	139	95	56
Surgical_units	293	227	171	139	93
Mean surgical unit volume	19 (10.2–29.1)	24 (14.1–34.9)	26.9 (18.8–36)	34.1 (21.5–55.8)	66.4 (32.6–118.2)
Age (years)					
<60	313 (18.7%)	344 (20.6%)	308 (18.4%)	338 (20.3%)	361 (21.6%)
60–64	229 (13.7%)	216 (12.9%)	205 (12.3%)	211 (12.6%)	195 (11.7%)
65–69	354 (21.2%)	326 (19.5%)	327 (19.6%)	330 (19.8%)	324 (19.4%)
70–74	328 (19.6%)	324 (19.4%)	353 (21.1%)	310 (18.6%)	322 (19.3%)
75–79	251 (15%)	244 (14.6%)	262 (15.7%)	279 (16.7%)	257 (15.4%)
80 or more	195 (11.7%)	216 (12.9%)	215 (12.9%)	201 (12%)	210 (12.6%)
Gender					
Female	987 (59.1%)	961 (57.5%)	970 (58.1%)	960 (57.5%)	936 (56.1%)
ASA grade					
ASA1	108 (6.5%)	104 (6.2%)	106 (6.3%)	117 (7%)	112 (6.7%)
ASA2	1150 (68.9%)	1136 (68%)	1150 (68.9%)	1143 (68.5%)	1161 (69.6%)
ASA3	412 (24.7%)	430 (25.7%)	414 (24.8%)	409 (24.5%)	396 (23.7%)
Year					
2009	111 (6.6%)	81 (4.9%)	69 (4.1%)	77 (4.6%)	99 (5.9%)
2010	123 (7.4%)	112 (6.7%)	115 (6.9%)	101 (6.1%)	89 (5.3%)
2011	142 (8.5%)	104 (6.2%)	92 (5.5%)	118 (7.1%)	81 (4.9%)
2012	165 (9.9%)	161 (9.6%)	132 (7.9%)	139 (8.3%)	132 (7.9%)
2013	180 (10.8%)	148 (8.9%)	125 (7.5%)	145 (8.7%)	154 (9.2%)
2014	162 (9.7%)	141 (8.4%)	154 (9.2%)	163 (9.8%)	151 (9%)
2015	166 (9.9%)	194 (11.6%)	205 (12.3%)	172 (10.3%)	160 (9.6%)
2016	155 (9.3%)	196 (11.7%)	193 (11.6%)	210 (12.6%)	189 (11.3%)
2017	207 (12.4%)	190 (11.4%)	243 (14.6%)	208 (12.5%)	255 (15.3%)
2018	163 (9.8%)	220 (13.2%)	229 (13.7%)	213 (12.8%)	231 (13.8%)
2019	96 (5.7%)	123 (7.4%)	113 (6.8%)	123 (7.4%)	128 (7.7%)
Operation funder					
Public	1530 (91.6%)	1551 (92.9%)	1550 (92.8%)	1527 (91.5%)	1513 (90.7%)
Private	140 (8.4%)	119 (7.1%)	120 (7.2%)	142 (8.5%)	156 (9.3%)

*Note*: Patient level descriptive statistics for single stage first revision total knee replacements for aseptic indications.

Abbreviation: ASA, American Society of Anaesthesiologists.

**Figure 2:**
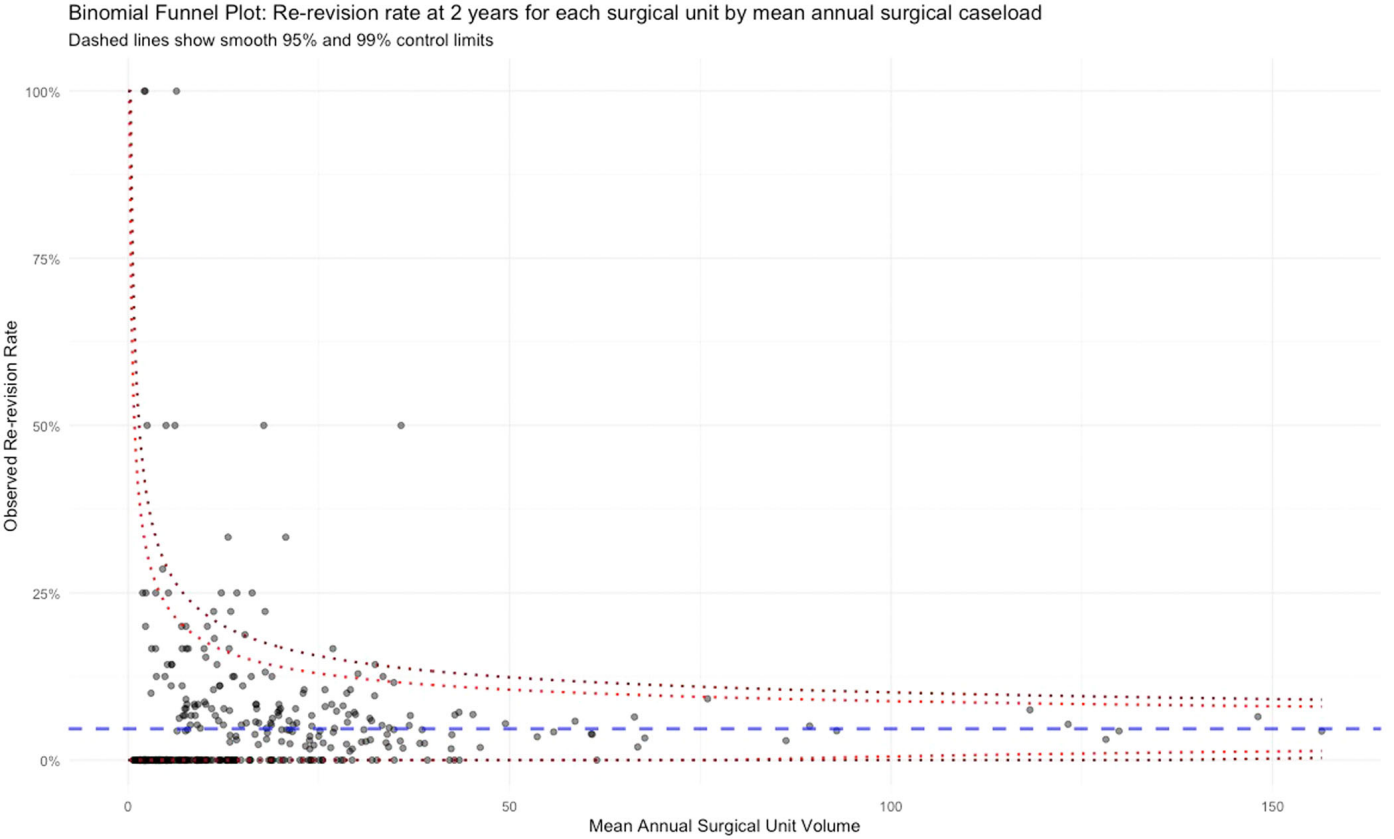
Binomial funnel plot showing re‐revision rates at 2 years for surgical units based on mean annual surgical unit volumes (dashed lines show 95% and 99% control limits).

**Figure 3:**
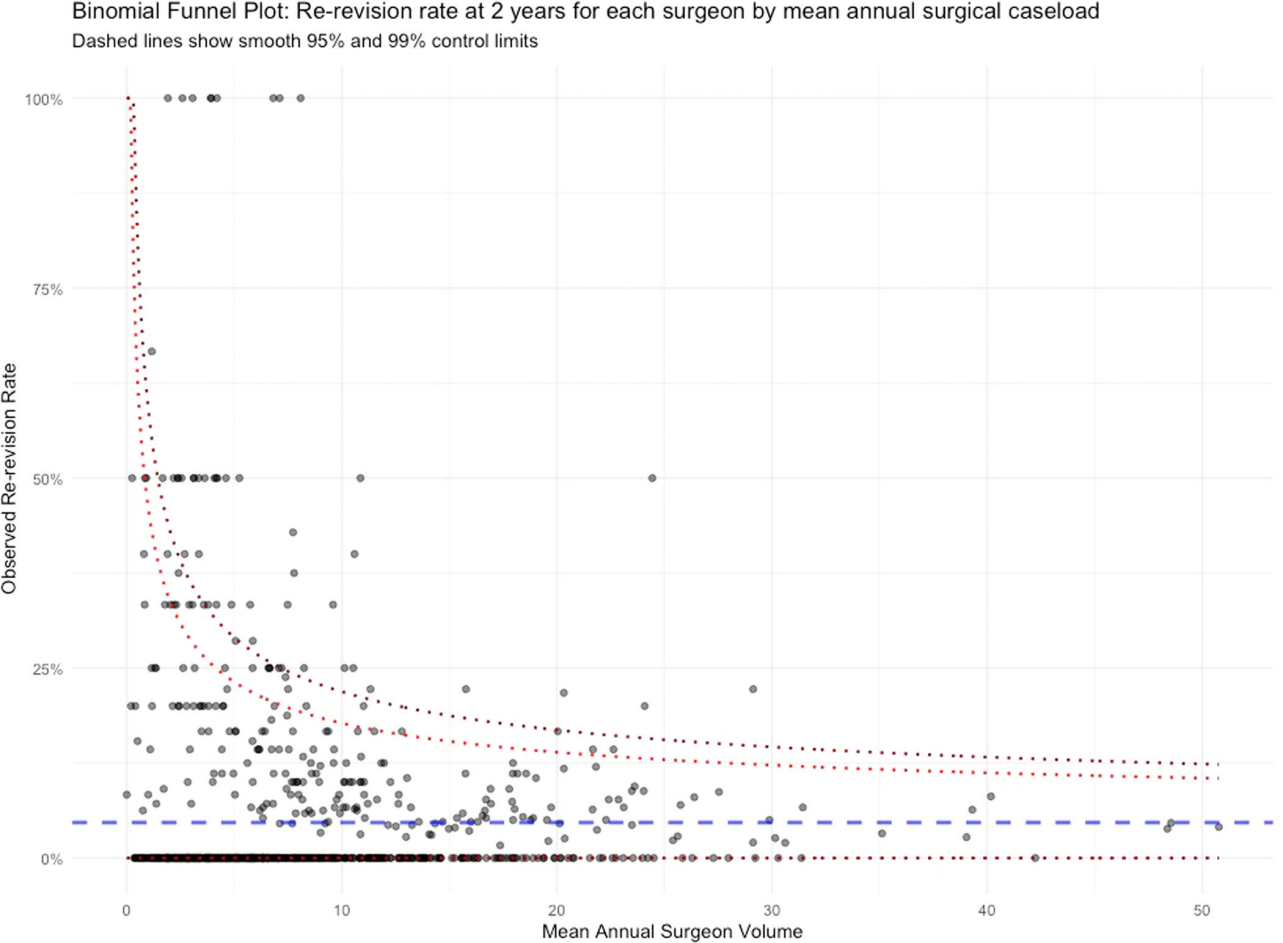
Binomial funnel plot showing re‐revision rates at 2 years for surgeons based on mean annual surgeon volumes (dashed lines show 95% and 99% control limits).

Only 407/7019 (5.8%) of cases underwent a re‐revision within 2 years. Figures 2 and 3 represent binomial funnel plots showing the unadjusted re‐revision rates at 2 years across a range of mean annual procedure volumes for units and surgeons respectively. There was no statistically significant association between unit or surgeon volume and outlier status (Spearman's *r* = −0.046, *p* = 0.37 for units; Spearman's *r* = −0.034, *p* = 0.31 for surgeons).

**Figure 4:**
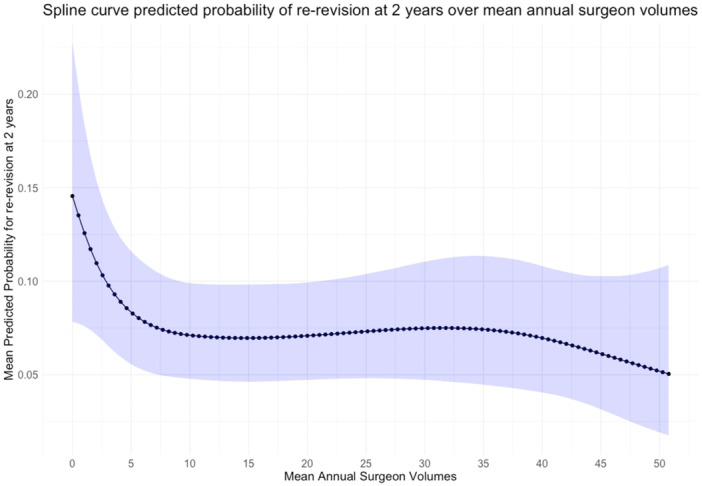
Predicted probability of re‐revision at 2 years by mean annual surgeon volume for single stage revision knee replacement for non‐infected causes. A fixed effects multivariable logistic regression model using three knots at 5%, 50% and 95% centiles of mean surgeon volume. 95% confidence intervals represented by blue shaded line.

Following modelling and adjustment of co‐variates, the odds of re‐revision decreased with increasing mean annual surgeon volume (Figure 4). A change point analysis detected a change at mean surgeon volume of ≥ 9 cases. Subsequent threshold categorisation and modelling of surgeon volume ≥9 cases demonstrated statistically significant lower rates of re‐revision (OR = 0.77 (95% CI 0.62–0.95, *p* = 0.02). The trend was not seen for mean surgical unit volumes. Please refer to Table 3 for adjusted outcomes for surgical unit volume and Table 4 for adjusted outcomes for surgeon volume.

**Table 3:** Multivariable logistic regression modelling of primary and secondary outcomes with mean surgical unit volume.

	Association between mean surgical unit volume (continuous) and secondary outcomes		
	Odds ratio¡/coefficient estimate• (95% confidence intervals)	*p* value	*R* ^2^
Primary outcomes			
Re‐revision within 2 years	1.00 (1.00–1.01)	0.08	6.9%
Secondary outcomes			
Any medical complication within 90 days	Supporting Information: Material S1	NA	8.3%
Mortality within 90 days	1.00 (0.99 to 1.01)	0.33	13.4%
Prolonged length of stay >5 days	Supporting Information: Material S2	NA	13.4%

**Table 4:** Multivariable logistic regression modelling of primary and secondary outcomes with mean surgeon volume.

	Association between mean surgeon volume (continuous) and secondary outcomes		
	Odds ratio¡/coefficient estimate• (95% confidence intervals)	*p* value	*R* ^2^
Primary outcomes			
Re‐revision within 2 years (>8 RevKR)	0.77 (0.62–0.95)	<0.05	6.7%
Secondary outcomes			
Any medical complication within 90 days	1.00 (1.00–1.01)	0.75	8.3%
Mortality within 90 days	1.00 (1.00–1.03)	0.84	13.4%
Prolonged length of stay >5 days	0.92 (0.85–0.99)	<0.05	13.4%

The risk of medical complications occurring within the first 90 days post‐operatively was higher for high volume surgical units (Supporting Information: Material S1). Higher volume units were more likely to manage patients with longer inpatient stays (Supporting Information: Material S2).

## DISCUSSION

The main finding of this study is higher volume surgeons have lower re‐revision rates within 2 years compared with lower volume surgeons. This association appears to be independent of known confounding factors. Increasing annual surgeon volume of 9 cases or more is significantly associated with a reduction in the odds of re‐revision within 2 years. The same effect was not observed when testing the association between hospital volume and re‐revision.

Comparison with previous literature is challenging due to the limited number of similar size registry‐based studies which report adjusted estimates. In a cohort of 169 patients, Roof et al. [20] found a lower re‐revision rate in surgeons performing more than 18 annual RevKR, 7.1% versus 19.0% respectively. The results in this study suggest first time revision for non‐infected reasons should be done by surgeons performing a minimum of nine annual revisions. This number is currently below the recommended minimum annual threshold of 15 per surgeon in England for all revisions including those for infection [4].

Our finding of no association between unit volume and re‐revision rates is supported by evidence from a large population study reporting data on 8072 patients from the Danish Joint Registry [28]. However, our results contrast with those reported by Halder et al. [10] involving 23,644 patients. Despite this study performing an adjusted analysis for known confounders, there was moderate selection bias using an administrative data set only representing approximately 30% of the German health population.

Analysis of secondary outcomes found higher surgical unit volume was associated with higher rates of prolonged hospital stay. This was a similar relationship observed by Lindberg‐Larsen et al. [14], who reported shorter length of stays in lower volume units following an adjusted analysis. This could indicate that higher volume surgical units are more likely to accept referrals of more complex cases and the measurement of this complexity is not collected within our data sets [25]. Conversely, higher surgeon volume was associated with statistically lower rates of prolonged hospital stays. These findings contrast those of Roof et al. [20] who observed no difference between higher surgeon volume and length of stay. However, their results were based on a small population in a single centre and their estimates were not adjusted for any known confounder.

Our study uses data from secondary data sources and as such has limitations common to observational research. Despite this, the NJR is a mature registry, one of the largest in the world and has been shown to have excellent data quality [23]. The reporting of data to the NJR became mandatory on 1 April 2011 and after this period should be a good approximation of the true re‐revision rate. However, patients not consenting to the NJR audit were omitted from this study potentially introducing a selection bias. It is estimated less than 10% of patients do not give consent to the NJR [19]. All publicly funded hospital admissions both in NHS and independent hospitals are recorded in HES. Financial incentives exist to improve the coding accuracy within national administrative data sets such as HES [11]. Linked HES and national mortality statistics have been shown to provide accurate and clinically useful predictions for mortality up to 1 year [1].

The relationship between volume and outcome, particularly within complex joint arthroplasty, is challenging to determine, particularly when using observational data repositories such as NJR and HES. This is because we have used a broad definition of a RevKR and the technical difficulty of procedures which fall under this definition is variable. The data set in this study was minimised to include only first‐time single stage revisions for non‐infected reasons (aseptic loosening/lysis, instability, component wear and malalignment) to help control for this and give as homogenous a sample as possible. A hierarchical classification was used to define the primary reason for revision, and this may oversimplify cases with multiple contributing factors. The complexity of the case mix is likely to vary with hospital volume [25].

Our exposure variable was calculated from all RevKR procedures and not limited to our final patient cohort. We acknowledge this could introduce potential inaccuracies as they were excluded from outcome analysis. However, we believe inclusion of these cases in caseload calculations is more representative of RevKR surgical experience particularly when overall RevKR now inform specific minimal volume targets for units and surgeons in England [4]. We acknowledge the surgical caseload calculation does not accommodate changes in unit volume between seasons or define changes in staff turnover. These factors are not addressed in the scope of this work and are a limitation.

The biggest limitation of this study is data omission. The data set used in this study is historical and this may affect the generalisability of the results as we preclude more recent data. However, this was intentional to avoid the substantial impact on revision volumes due to the pandemic seen in 2020–2021 [21]. Of all recorded first time RevKR, 26.1% (8695/33,282) were included in the final analysis. We recorded a very high record attrition of completed PROMs data with only 22.5% (1948/8695) of our population recording complete pre and post‐operative PROMs. This precluded any meaningful analysis of PROMs. One of the disadvantages of using revision as marker of success is that the decision to revise a prosthesis depends on many factors. The inclusion of pre‐operative PROMs would have helped to differentiate the revision thresholds between different volume surgeons. We acknowledge this limitation of the study.

In RevKR for infection it has been noted that higher volume units are associated with better re‐revision rates at 2 years [15]. The aim of this work was to create a model for less complex revisions, and in this cohort, it was found that higher surgeon volume is linked to better re‐revision rates at 2 years. Surgeons performing nine or more revisions can expect to get better outcomes and subsequently all revision surgeons should be auditing their practice to ensure this target is met. Unit volume appears to have a more complicated relationship with outcomes, and this merits further investigation.

As part of the revision knee network programme in England, outlier units and surgeons should be highlighted, and data examined in detail on the role of case mix or lack of multidisciplinary network engagement as potential influences on outcome. The absence of re‐revision outliers in higher volume units infer that it may be more pragmatic for these procedures to be carried out by higher volume centres with the intention of reducing the number of outlier units. It may also be more cost effective if RevKR was performed by fewer centres from a cost saving approach, as higher volume centres have been found to have lower costs in revision hip and knee replacement surgery [9].

## CONCLUSION

We provide evidence of an association between the number of RevKRs a surgeon performs and re‐revision rates within 2 years. Increasing surgeon annual volume of nine or over was independently associated with a reduction in the odds of revision.

## AUTHOR CONTRIBUTIONS

**Alexander H. Matthews**: Conceptualisation; methodology; funding acquisition; software; project administration; investigation; data curation; formal analysis; validation; visualisation; writing–original draft; writing–review and editing. **William K. Gray**: Conceptualisation; methodology; investigation; validation; supervision; writing–review and editing. **Jonathan P. Evans**: Conceptualisation; supervision; writing–review and editing. **Jonathan T. Evans**: Supervision; writing–review and editing. **Sarah E. Lamb**: Supervision; writing–review and editing. **Andrew Porteous**: Funding acquisition; writing–review and editing. **Timothy Briggs**: Funding acquisition; supervision; writing–review and editing. **Shiraz Sabah**: Conceptualisation; data curation; validation; writing–review and editing; supervision. **Abtin Alvand**: Supervision; writing–review and editing. **Andrew Toms**: Conceptualisation; funding acquisition; supervision; writing–review and editing. **Andrew Price**: Conceptualisation; funding acquisition; supervision; writing–review and editing.

## CONFLICT OF INTEREST STATEMENT

The authors declare no conflicts of interest.

## ETHICS STATEMENT

Ethical approval was obtained from the London‐Bromley Research Ethics Committee (20/LO/0428). Data access approvals were obtained from the National Joint Registry for England, Wales, Northern Ireland, the Isle of Man and the States of Guernsey (NJR) (RSC2017/26). Patients who did not consent to the NJR audit were not included.

## Supporting information

Supporting information.

Supporting information.

Supporting information.

## Data Availability

The data sets generated and analysed in the current study are not publicly available due to data protection regulations. Access to data is limited to the researchers who have obtained permission for data processing. Further inquiries can be made to the National Joint Registry research team.
