# Supplementary material for: Higher surgeon volume reduces early failure in first time revision of non‐infected total knee arthroplasty: An analysis using data from the United Kingdom National Joint Registry
Source: Knee Surg Sports Traumatol Arthrosc. 2025 May 12;33(9):3286–97. doi: 10.1002/ksa.12690 (PMC12392377; doi:10.1002/ksa.12690)

# Supplementary material S1 – Predicted probability of medical complications within 90 days by mean surgical unit volume

A Fixed effects multivariable logistic regression model using 3 knots at 5%, 50% and 95% centiles of mean unit volume and age. 95% confidence intervals represented by blue shaded line.


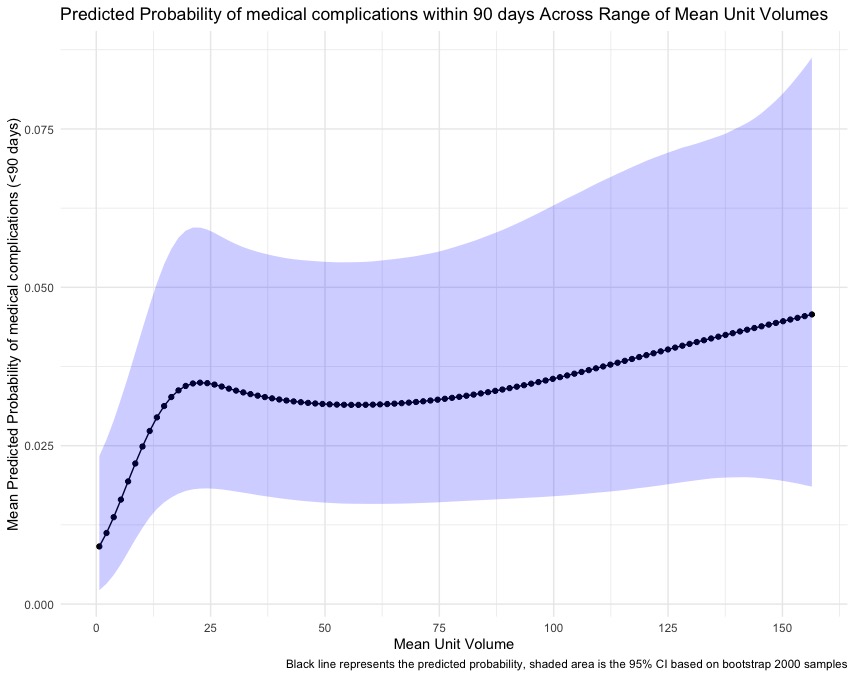


# Supplementary material S2 – The relationship between mean surgical unit volume and the predicted probability of length of stay (LOS) greater than 5 days

Adjusted multi-variable logistic regression model for mean surgical volume and rates of prolonged length of stay. Surgical volume was modelled using restricted cubic splines in view of its non-linear relationship with length of stay. 95% confidence intervals are represented by the shaded area.


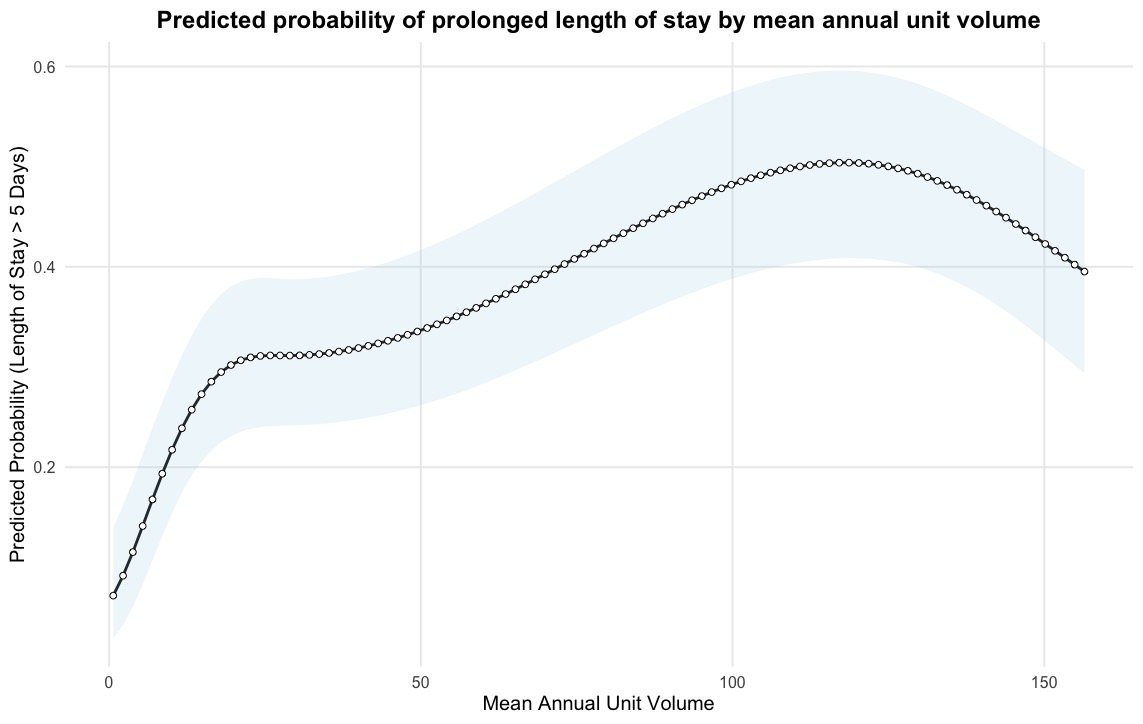

Supplement: Supplementary file 1 — Supporting information. [file KSA-33-3286-s003.docx]
