# Supplementary figures and images for: Higher surgeon volume reduces early failure in first time revision of non‐infected total knee arthroplasty: An analysis using data from the United Kingdom National Joint Registry
Source: Knee Surg Sports Traumatol Arthrosc. 2025 May 12;33(9):3286–97. doi: 10.1002/ksa.12690 (PMC12392377; doi:10.1002/ksa.12690)

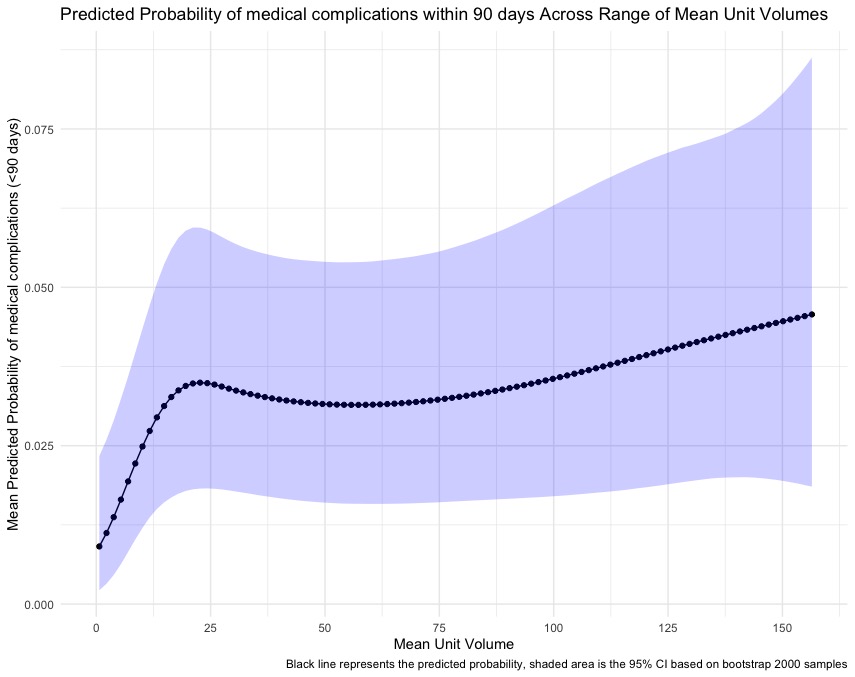

Supplement: Supplementary file 2 — Supporting information. [file KSA-33-3286-s001.jpeg]

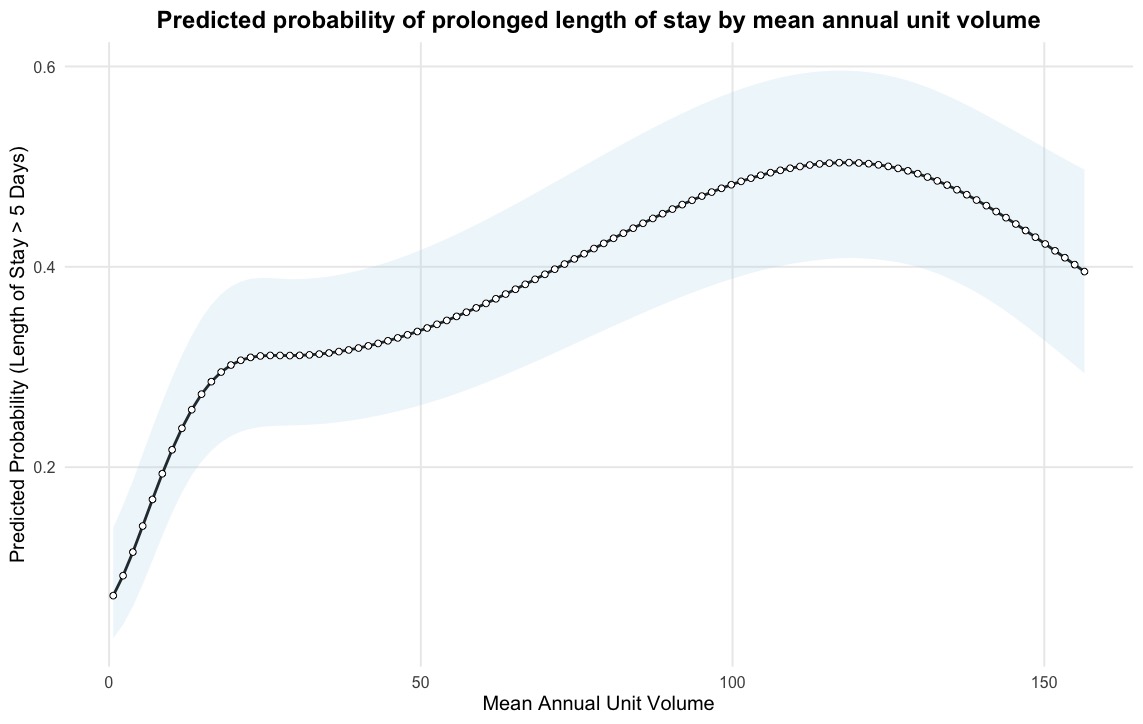

Supplement: Supplementary file 3 — Supporting information. [file KSA-33-3286-s002.jpeg]
